# Time Perspective Prediction Based on Early Maladaptive Schemas Mediated by Life Expectancy Among University Students: A Cross‐Sectional Study

**DOI:** 10.1002/hsr2.70924

**Published:** 2025-06-17

**Authors:** Mohammad Saeed Abedi Yarandi, Mohammad Ebrahim Madahi

**Affiliations:** ^1^ Department of Psychology, Faculty of Humanities Shahed University Tehran Iran

**Keywords:** early maladaptive schemas, life expectancy, time perspective

## Abstract

**Background and Aims:**

Time perspective is a fundamental component of individual cognition, often conceptualized through one's perception of time. Childhood experiences play a pivotal role in shaping an individual's temporal outlook. This study aims to investigate the predictive relationship between early maladaptive schemas and the five dimensions of time perspective, with life expectancy as a mediating variable.

**Methods:**

This cross‐sectional study included 336 students (43.45% male, 56.55% female) from Shahed University, selected through convenience sampling. Participants were drawn from various faculties, and both genders were represented proportionately. Data collection occurred in June 2023, utilizing the Young Schema Questionnaire Short Form, Snyder's Hope Scale, and the Zimbardo Time Perspective Inventory. Data analysis was conducted using SPSS version 25 and Amos version 24.

**Results:**

Data analysis reveals that the correlation matrix shows significant associations (*p* < 0.001) between most early maladaptive schema domains and the majority of time perspective subscales. Additionally, early maladaptive schemas directly predict the negative past, present hedonistic, and present deterministic dimensions of time perspective. Meanwhile, the positive past, present hedonistic, present deterministic, and future dimensions are indirectly predicted through the mediating effect of life expectancy. Fit indices (CFI > 0.9, GFI > 0.9, IFI > 0.9, AGFI > 0.8) indicate a strong model fit.

**Conclusion:**

The findings indicate that Young's schemas are significant predictors of time perspective. These results highlight the need to reconsider parenting practices, as they are a primary source of schema formation. By addressing maladaptive schema development in childhood, individuals may develop healthier time perspectives.

## 1 Background

This study was conducted among 336 students in Shahed University in Tehran, Iran. The choice of this university was because the authors were in this university and it was possible to collect data accurately among the students of this university. We examined time perspective, early maladaptive schemas, and life expectancy in these students. The purpose of this study is to predict time perspective based on early maladaptive schemas with the mediation of life expectancy.

Time has been and continues to be an essential concept throughout every individual's life and the history of humanity. Time is a concept that individuals always live with, gaining greater importance due to the rapid changes in our surrounding world, especially in this era. Therefore, from various perspectives, time has been under examination. One of these dimensions in psychology is the concept of time perspective. Time perspective, first introduced in 1938 by Lawrence Frank [1], follows individuals’ views of their psychological future and past at a specific time. Later, in the late twentieth century, Zimbardo studied the concept of time [2]. Time perspective is a preferential direction of an individual's thoughts toward the past, present, or future [3]. Or whether a person thinks more about the past, present or future. Zimbardo's theory combined motivational, emotional, cognitive, and social processes. According to McKeown's definition [2], time perspective is an intrusive influence that past, present, and future considerations have on a wide range of human behaviors. Time perspective shows how people view time. It can be said that the personal feeling of each of us towards time is one of the strongest factors that affect our thoughts, feelings and actions. All of us humans live in three mental areas: the past; It means something that was… now; It means what is… and the future; It means what will come… Each of these boundaries has taken the color of cultural prejudices, spiritual beliefs, memories and thousands of hopes, dreams and life experiences [4, 5]. Boyd and Zimbardo stated that healthy psychological functioning is associated with balancing the three temporal dimensions of past, present, and future [6]. This balance can reflect an individual's abilities in learning from the past, adapting to the present, and preparing for goal‐oriented behaviors in the future. Time perspective has five dimensions: positive past, negative past, present hedonistic, present deterministic, and future [4]. Time perspective is a crucial variable in our life and it has various effects on our life. Some time perspectives predict future marital dissatisfaction [7]. The utility of time perspective, exceptionally balanced time perspective patterns, plays an influential and significant role in career growth applications [8]. Time perspective predicts positive and negative academic emotions in students [9]. Adolescents’ future time perspective may serve as a protective factor for academic achievement [10]. Time perspective dimensions significantly predict academic dishonesty through self‐regulatory variables [11]. Balanced time perspective could predict lower levels of aggression in left‐behind children [12]. Some time perspectives predict marital instability [13]. Some predict loneliness [14]. Some predict hopefulness [15]. These, along with other research studies, underscore the importance of time perspective.

Time perspective may be influenced by an individual's past [16]. Even later in life, Adolescents from more functional families exhibit a higher degree of balanced time perspective [17]. It is possible that based on real experiences of undesirable and harmful events, the individual may now have a specific time perspective. Therefore, examining an individual's early schemas becomes highly significant. Because it originates from a person's past.

Schema is generally defined as a structure, framework, or template with a rich and eminent history in psychology, particularly in the cognitive domain. Young believes that some of these schemas may form the core of personality disorders, milder cognitive dysfunctions, and many Axis I chronic disorders. To further examine this idea, Young has identified a set of schemas, which he calls early maladaptive schemas. Young defines early maladaptive schemas as self‐defeating emotional and cognitive patterns that form early in development and repeat throughout life. Early maladaptive schemas are fixed and repetitive incorrect patterns formed in a person's mind. These schemas manifest as themes of disconnection and rejection, impaired autonomy and performance, impaired limits, other‐directedness, and over‐vigilance and inhibition [18].

To examine the relationship between early maladaptive schemas and time perspective at a theoretical level and based on existing studies in this field, we delve into some of Young's theories and definitions of early maladaptive schemas, as well as studies on time perspective and definitions of some of its dimensions.

For example, some early maladaptive schemas, such as abandonment/instability, mistrust/abuse, emotional deprivation, defectiveness/shame, and social isolation/alienation, all of which fall under the domain of disconnection and rejection. According to Young, these schemas are observed in individuals who anticipate that their basic needs will not be predictably met [18]. This anticipation is based on the individual's outlook and experience in the past. Individuals develop these schemas based on negative past experiences they have had in life [18]. This concept is closely related to the idea of the negative time perspective. A negative time perspective is “a hostile and distressing view of the past that may result from the actual experience of undesirable and harmful events or from the negative reconstruction of harmless events or a combination of both” [19].

Another example of early maladaptive schemas is those defined as “self‐defeating emotional and cognitive patterns. They form early in development and repeat throughout life” [18], which contrasts conceptually with the positive past time perspective, reflecting “a warm and emotional attitude toward the past” [19]. Despite the theoretical relationship between early maladaptive schemas and time perspective, there is a noticeable lack of research in this field and almost no research was found that deals with the relationship between these two variables.

Hope is another variable of this study that has a mediating role. Hope is the ability to imagine the ability to create paths towards desirable goals in the future and to imagine having the motivation to move along these paths [20]. Regarding the relationship between early maladaptive schemas and life expectancy, it should be noted that these schemas, alongside their damages, can also impact our life expectancy. Studies have examined the relationship between life expectancy and early maladaptive schemas. These studies have found a significant relationship between some of these schemas, such as rejection and abandonment, and hopefulness [21]. In addition, according to Seligman's theory, Young's schemas can affect an individual's mental resilience and adaptive coping strategies. It has been suggested that addressing and modifying these schemas can improve hopefulness and resilience [22]. Schema therapy has been effective in increasing life expectancy [23]. From a theoretical standpoint, schemas in which the individual anticipates that their basic needs will not be met [18] can conflict with life expectancy, which is considered one of the coping resources for humans in dealing with problems and even incurable diseases [24]. Furthermore, this hope can be described as a multidimensional, dynamic, and powerful healing factor and can play a significant role in coping with loss [25]. Naturally, there is a conflict in this concept.

Regarding the relationship between time perspective and life expectancy, it should be noted that in a study, the role of hopefulness in predicting dimensions of time perspective has been confirmed. The results of this study have shown that hopefulness can positively predict the present hedonistic and future time perspectives while negatively predicting the negative past and present deterministic time perspectives [26]. Theoretically, the future time perspective dimension is defined as “a general orientation toward the future involving control behavior through efforts to achieve future goals and rewards” [19], which aligns with the concept of life expectancy.

The concept of time perspective has been investigated in various studies. For example, in one study, hope has been predicted based on time perspective [15], while in another study, the mediating role of life expectancy between psychological distress and time perspective has been examined [27].

In this study, we want to investigate one of the factors that play a role in the emergence of time perspective dimensions. As mentioned, early maladaptive schemas theoretically predict time perspective well, but so far no research has predicted time perspective and there is little study in the field of time perspective. This study is the first research that introduces us a variable that can predict the time perspective so that we can correct it and move towards a balanced perspective.

## 2 Materials and Methods

### 2.1 Research Questions

1. Are early maladaptive schemas predictors of dimensions of time perspective?
2. Does life expectancy mediate in predicting time perspective based on early maladaptive schemas?

### 2.2 Research Design

Given the nature of the subject, a descriptive study of the relationships between variables, structural equation model, was conducted. In this study, early maladaptive schemas were considered as independent variables, time perspective as the dependent variable, and life expectancy as a mediating variable.

### 2.3 Sampling Method

There is no consensus on the optimal sample size for such research. Based on Jackson's research, about 20 samples are needed for each factor (latent variable) in structural equation modeling [28]. In SEM analysis, the required sample of at least 10 times the number of observed variables is considered as a general rule. A more realistic minimum sample size is based on the number of latent variables and the number of variables measured, and to identify the standard effect size, the expected coefficients are estimated at 95% confidence level and 80% test power [29]. In this sample size design, an analytic calculator software was used. The required sample size was estimated at 316 samples to identify the expected effect size of 0.17 with 2 latent variables and 12 observed variables that were measured in the SEM model with 95% confidence level and 80% test power [30].

Due to the lack of a list of students and the lack of university cooperation and the large number of samples, we had to use the convenience sampling method. But It was tried to respect the ratio of faculties in the research and to be in accordance with the population. Additionally, to compare the characteristics of the sample with the characteristics of the population, a chi‐square test was used for demographic variables.

A cross‐sectional study was conducted with 336 students (45.43% male, 55.56% female) enrolled at Shahed University, located in Tehran, Iran, in June 2023. All participants were native Persian speakers.

### 2.4 Data Collection Instruments

The data collection instruments comprised of three items:

- Time Perspective Inventory: Developed by Zimbardo and Boyd in 1999, consists of 56 items divided into five dimensions, with responses ranging from “very false” (score of 1) to “very true” (score of 5) for each item. Higher scores on each dimension indicate a predominance of that particular time perspective. The developers evaluated the Inventory in 26 samples from 24 countries and reported acceptable reliability and validity. Fit indices, including RMSEA = 0.057, SRMR = 0.062, and CFI = 0.086, indicated a satisfactory model fit [5]. By studying 866 people in Iran, this Inventory showed that 42 of the 56 items belong to their appropriate factors and 8 items had cross‐loadings over 0.30 on inappropriate factors. A total of 4 items did not show a substantial loading (> 0.30) on any of the factors and at least 20 items emerged with factor loadings less than 0.40 [31].
- Early Maladaptive Schemas Questionnaire: Developed by Young in 1988, this questionnaire comprises 75 items to assess 15 early maladaptive schemas. Respondents rate each item on a Likert scale ranging from “completely disagree” to “completely agree.” Young based this questionnaire on its original version, which included 205 items. Smith et al. (1995) reported a Cronbach's alpha reliability coefficient of 0.96 for the entire questionnaire and above 0.80 for its subscales [32]. Standardization of this questionnaire was carried out in Iran by Ahi in 2005 at Tehran universities, where it demonstrated good validity and reliability [33].
- Hope Scale Questionnaire: Developed by Snyder and colleagues, this questionnaire consists of 12 items designed to assess individuals’ level of life expectancy. The scoring method is based on an 8‐point Likert scale, ranging from “completely disagree” to “completely agree.” The total score ranges from 1 to 8. Subscales include pathways thinking and agency thinking, each comprising four items. In a study involving 342 participants aged 18‐21, the reliability coefficient for pathway thinking was 0.76, and for agency thinking, it was 0.75 [34]. In Iran, the necessary validity was confirmed through Cronbach's alpha [35, 36].

It should be noted that all participants participated in this study with their full consent and answered the questionnaires. All personal information will be kept confidential.

### 2.5 Data Analysis

In this study, the collected data were analyzed and interpreted using various methods such as descriptive statistics (mean, standard deviation, frequency, etc.) and inferential statistics (correlation coefficient and structural equation modeling). Confirmatory factor analysis was employed as the method for validation, utilizing techniques such as factor analysis, confirmatory factor analysis, and path analysis. In the structural equation model, confirmatory factor analysis was initially conducted for the two questionnaires, Early Maladaptive Schemas and Hope Scale, which served as indicators. Then, the model was evaluated. In this study, direct and indirect path coefficients between the latent variables in the structural model were measured. The confidence level is 95%. The analysis was performed using SPSS 25 and Amos 24 software.

## 3 Results

From a total of 336 participants in the study, 136 individuals (43.45%) were male. The ratio of the number of students in each faculty was observed in the sampling to increase accuracy.

Table 1 presents the demographic characteristics of the participants. Each demographic characteristic is represented separately, and their frequency is indicated. Descriptive statistics are provided in Table 2, and the correlation matrix is shown in Table 3.

**Table 1:** Frequency and percentage of students in faculties.

	Population			Sample		
SubjectsFaculty	Frequency	Percentage	Cumulative percentage	Frequency	Percentage	Cumulative percentage
Nursing and Midwifery	195	4	4	10	3	3
Medicine	409	7	11	31	9	12
Dental	176	3	14	10	3	15
Humanities	2160	38	52	136	40	55
Basic Sciences	710	13	65	33	10	65
Agricultural Sciences	172	3	68	10	3	68
Engineering	1368	24	82	75	23	91
Art	437	8	100	31	9	100
Total	5627	100	—	336	100	—

**Table 2:** Descriptive indicators of variables.

Variable	Mean	Median	Std.	Skewness	Kurtosis	α Cronbach's alpha
Negative Past Perspective	3.41	3.40	0.66	−0.23	**−0.09**	0.78
Positive Past Perspective	3.28	3.33	0.48	−0.12	0.04	0.51
Deterministic Present Perspective	2.86	2.83	0.66	0.18	−0.50	0.73
Hedonistic Present Perspective	3.24	3.27	0.47	0.41	0.21	0.68
Future Perspective	3.57	3.58	0.54	−0.15	−0.05	0.72
Overvigilance Inhibition	35.43	35.50	10.41	−0.02	−0.31	0.84
Other Directedness	28.89	28.00	9.33	0.44	−0.06	0.82
Impaired Limits	33.46	34.00	9.39	−0.02	−0.20	0.79
Impaired Autonomy And Performance	44.20	40.00	18.73	1.02	0.74	0.92
Disconnection And Rejection	68.62	66.50	22.46	0.34	−0.40	0.92
Active thinking	22.81	23.00	5.29	−0.40	−0.22	0.80
Path	23.27	24.00	4.67	−0.47	0.09	0.65

**Table 3:** Correlation matrix of variables.

Variables	1	2	3	4	5	6	7	8	9	10	11	12
1. Time perspective ‐ Negative past	_											
2. Time perspective ‐ Positive past	−0.092	_										
3. Time perspective ‐ Deterministic present	0.442**	−0.037	_									
4. Time perspective ‐ Hedonistic present	0.169**	0.173**	0.320**	_								
5. Time perspective – Future	−0.044	0.193**	−0.317**	−0.168**	_							
6. Schema ‐ Disconnection And Rejection	0.503**	−0.237**	0.399**	0.005	−0.228**	_						
7. Schema ‐ Impaired Autonomy And Performance	0.505**	−0.068	0.476**	0.076	−0.223**	0.726**	_					
8. Schema – ImpairedLimits	0.464**	−0.092	0.335**	0.294**	−0.248**	0.544**	0.500**	_				
9. Schema – OtherDirectedness	0.509**	−0.070	0.358**	0.080	−0.171**	0.624**	0.618**	0.476**	_			
10. Schema ‐ Overvigilance Inhibition	0.451**	−0.136*	0.175**	−0.104	0.089	0.579**	0.468**	0.482**	0.479**	_		
11. Life expectancy ‐ Active thinking	−0.340**	0.319**	−0.378**	0.145**	0.511**	−0.439**	−0.509**	−0.292**	−0.347**	−0.250**	_	
12. Life expectancy – Path	−0.148**	0.207**	−0.380**	0.112*	0.395**	−0.283**	−0.402**	−0.136*	−0.206**	−0.130*	0.683**	_

* *p* < 0.05

** *p* < 0.01

In Table 2, α was greater than 0.5 in all subscales of the research variables, which indicates their good correlation and reliability.

The results of the Table 4 show that the issue of collinearity among the predictor variables of the research did not occur. Because the tolerance coefficient values are greater than 0.1 and the variance inflation factor values are less than 10 for each predictor variable [37].

**Table 4:** Collinearity Statistics.

Model	Collinearity Statistics	
	Tolerance	VIF
Schema ‐ Disconnection And Rejection	0.360	2.777
Schema ‐ Impaired Autonomy And Performance	0.376	2.661
Schema – Impaired Limits	0.627	1.595
Schema – Other Directedness	0.527	1.898
Schema – Over vigilance Inhibition	0.611	1.637
Life expectancy ‐ Active thinking	0.452	2.211
Life expectancy – Path	0.514	1.945

As can be seen in Table 3, the components of life expectancy have a significant relationship at the 0.01 level with most domains of early maladaptive schemas and time perspective subscales. Also, most domains of early maladaptive schemas have a significant relationship at the 0.01 level with most time perspective subscales.

Table 5 demonstrates that the standardized factor loadings of all indicators exceed 0.32. Factor loadings below 0.32 are considered weak and indicate that such indicators do not possess the necessary capacity to measure their respective latent variable effectively. Based on the results of Table 5, it can be concluded that all indicators meet the essential criteria for measuring their latent variable effectively.

**Table 5:** Parameters of the research measurement model in confirmatory factor analysis.

Variables	B Unstandardized coefficients	β Standardized coefficients	SE	C.R	*p* value
Schema ‐ Impaired Autonomy And Performance	0.77	0.81	0.04	17.29	0.001
Schema – Impaired Limits	0.3	0.64	0.02	12.48	0.001
Schema – Other Directedness	0.35	0.73	0.02	15.04	0.001
Schema ‐ Overvigilance Inhibition	0.34	0.65	0.02	12.70	0.001
Schema ‐ Disconnection And Rejection	1	0.88			
Life expectancy (Active thinking) ‐ item 2	1.44	0.77	0.12	11.54	0.00
Life expectancy (Active thinking) ‐ item 9	1.20	0.70	0.10	11.17	0.00
Life expectancy (Active thinking) ‐ item 10	1.30	0.73	0.12	12.01	0.00
Life expectancy (Active thinking) ‐ item 12	1.00	0.68			
Life expectancy (Path) ‐ item 4	1.14	0.67	0.12	9.05	0.00
Life expectancy (Path) ‐ item 7	0.52	0.28	0.16	4.52	0.00
Life expectancy (Path) ‐ item 8	1.59	0.84	0.15	10.06	0.00
Life expectancy (Path) ‐ item 1	1.00	0.57			

Table 5 indicates that the standardized factor loadings of all indicators except item 7 are above 0.32. Factor loadings below 0.32 are considered weak, suggesting that such indicators do not have the necessary potential to measure their corresponding latent variable. However, since item 7 is significant, there is no need to remove this indicator. Based on the results of Table 5, it can be concluded that all indicators have the necessary capability to measure their latent variable.

Based on the goodness‐of‐fit indices resulting from confirmatory factor analysis in evaluating the measurement model of the study, it was concluded that the observed variables likely possess the necessary capacity to measure their corresponding latent variable (early maladaptive schemas). Criterion of fit indices: RMSEA < 0.08, CMIN < 3, CFI > 0.9, GFI > 0.9, IFI > 0.9, AGFI < 0.08, TLI > 0.9. This conclusion is drawn because at least 3 out of the fit indices need to be satisfactory or acceptable, which is the case as indicated in Table 6, where 7 out of 7 indices are deemed adequate.

**Table 6:** The Fit variables.

The fit of early maladaptive schemas							
Index of fit	RMSEA	CMIN (Chi‐square Minimum)	CFI	GFI	IFI	SRMR	TLI
Model	0.07	2.99	0.98	0.98	0.98	0.03	0.97
Criterion	< 0.08	< 3	> 0.9	> 0.9	> 0.9	< 0.08	> 0.9
Interpretation of Fit	Optimal	Optimal	Optimal	Optimal	Optimal	Optimal	Optimal
**The fit of life expectancy**							
Model	0.08	3.67	0.94	0.95	0.94	0.04	0.94
Criterion	< 0.08	< 3	> 0.9	> 0.9	> 0.9	< 0.08	> 0.9
Interpretation of Fit	Optimal	Un acceptable	Optimal	Optimal	Optimal	Optimal	Optimal
**First model fit**							
Model	0.13	7.13	0.82	0.85	0.83	0.08	0.76
Criterion	< 0.08	< 3	> 0.9	> 0.9	> 0.9	< 0.08	> 0.9
Interpretation of Fit	Un acceptable	Un acceptable	Un acceptable	Un acceptable	Un acceptable	Un acceptable	Un acceptable
**Modified model fit**							
Model	0.11	5.32	0.9	0.9	0.9	0.06	0.83
Criterion	< 0.08	< 3	> 0.9	> 0.9	> 0.9	< 0.08	> 0.9
Interpretation of Fit	Un acceptable	Un acceptable	Optimal	Optimal	Optimal	Optimal	Un acceptable

*Note:* Degrees of freedom early maladaptive schemas = 5

Degrees of freedom first model fit = 48

Degrees of freedom modified model fit = 39

Based on the goodness‐of‐fit indices obtained from confirmatory factor analysis in evaluating the measurement model of the study, it was concluded that the observed variables likely have the necessary potential to measure their corresponding latent variable (life expectancy). This is because a minimum of 3 of the fit indices need to be acceptable or desirable, and in Table 6, six fit indices are desirable.

**Figure 1:**
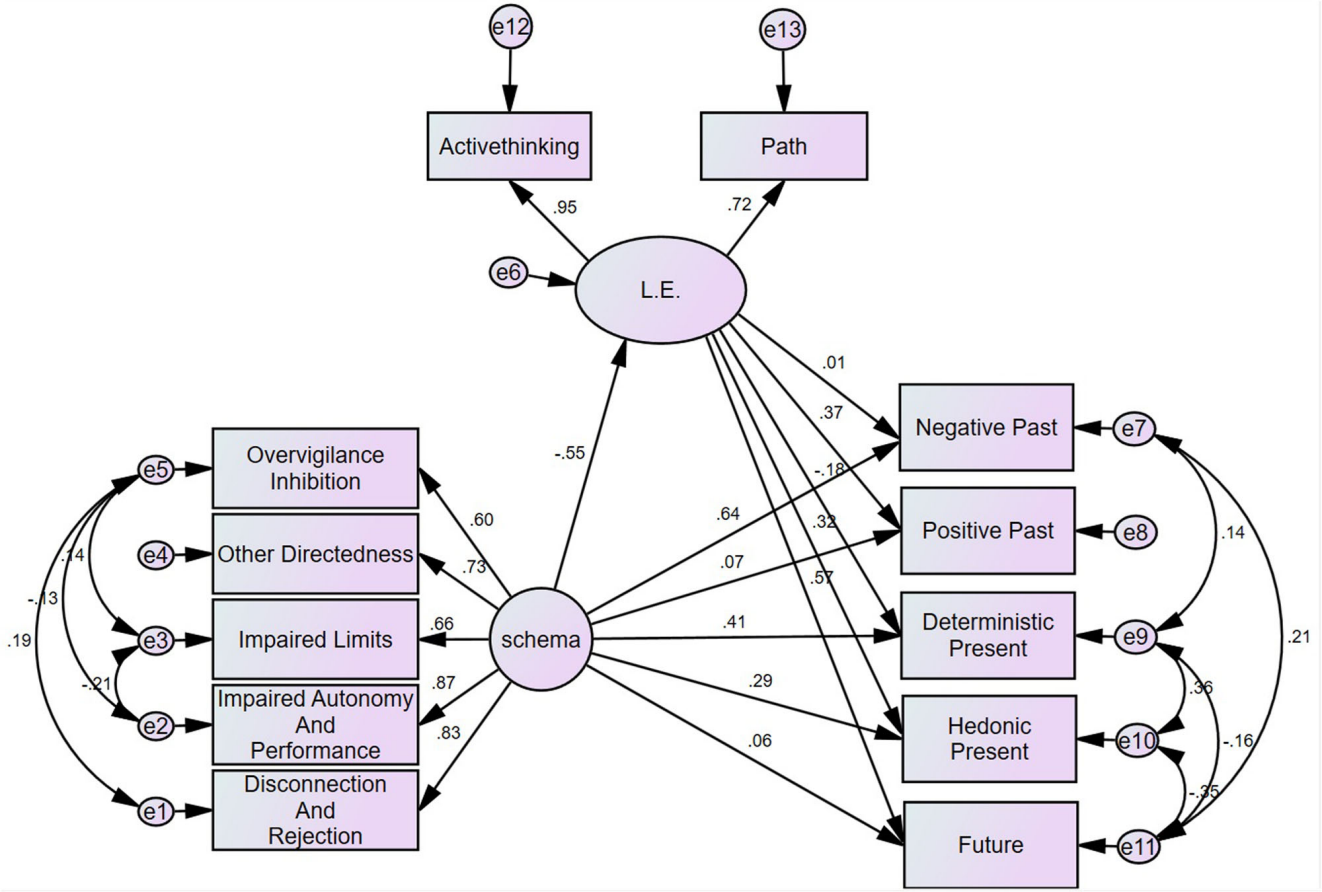
Structural relationships of early maladaptive schemas and life expectancy with time perspective in college students.

Based on Table 6, examining the fit indices resulting from the structural model test of the research showed that the fit indices do not support an acceptable fit of the model with the data. Therefore, modification indices were evaluated, indicating that by creating covariance between some indicator errors, the fit index could be improved. Accordingly, a modified model and the resulting fit indices were obtained, as presented in Table 6. Based on the fit indices resulting from the modified model, it was concluded that this model is satisfactory because at least 3 of the fit indices need to be desirable or acceptable, which is the case for the 4 indices in Table 6. Figure 1 shows the final model.

In Table 7, it is observed that early maladaptive schemas directly predict Negative Past dimensions (β = 0.640), Present Hedonistic (β = 0.295), Present Deterministic (β = 0.412), and Positive Past dimensions (β = −0.207), Present Hedonistic (β = −0.178), Present Deterministic (β = 0.101), and Future (β = −0.316) through the mediation of life expectancy.

**Table 7:** Direction coefficient of research variables.

Direct directions	B	β	SE	*p*	C.R.
Schema → Positive past	0.001	0.067	0.002	0.333	0.94
Schema → Negative past	0.023	0.64	0.002	0.001	10.05
Schema → Hedonistic present	0.008	0.295	0.002	0.001	3.91
Schema → Deterministic present	0.015	0.412	0.002	0.001	6.24
Schema → Future	0.002	0.063	0.002	0.33	0.94
Schema → Life expectancy	−0.149	−0.553	0.015	0.001	−9.67
Life expectancy → Negative past	0.002	0.015	0.008	0.802	0.24
Life expectancy → Positive past	0.036	0.374	0.007	0.001	5.24
Life expectancy → Deterministic present	−0.024	−0.182	0.008	0.004	−2.80
Life expectancy → Hedonistic present	0.03	0.321	0.007	0.001	4.34
Life expectancy → Future	0.062	0.572	0.008	0.001	7.94
Indirect directions	**B**	**β**	**SE**	***p***	**C.R.**
Schema → Positive past	−0.005	−0.207	0.045	0.006	−4.6
Schema → Negative past	0	−0.008	0.039	0.711	−0.20
Schema → Hedonistic present	−0.005	−0.178	0.049	0.009	−3.63
Schema → Deterministic present	0.004	0.101	0.043	0.012	2.34
Schema → Future	−0.009	−0.316	0.051	0.005	−6.19

The bootstrap method was used to examine the significance of indirect effects in the Amos software.

## 4 Discussion

Our research aimed to address two questions: Are early maladaptive schemas predictors of time perspective dimensions? And does life expectancy mediate in predicting time perspective based on early maladaptive schemas? Based on the results of this study, it can be stated that:

The early maladaptive schemas directly predict negative past. Naturally, domains such as Other‐Directedness, Over‐vigilance, and others are associated with negative past because schemas of other‐directedness typically arise in families where the child is accepted conditionally: the child must disregard essential aspects of their personality to gain love and acceptance from others. In many of these families, the emotional needs and desires of the parents and their social status are valued more than the needs and feelings of the children [16]. Early maladaptive schemas are negatively correlated with well‐being and family support [38]. Also, insecure attachment is positively correlated with early maladaptive schemas [39]. Maladaptive schemas of over‐vigilance typically develop in families where, instead of discipline, appropriate confrontation, reasonable limits, responsibility, cooperation, and goal‐setting, their characteristic aspect is excessive leniency, confusion, or a sense of superiority. In some cases, the child may be unable to tolerate normal distress or not receive sufficient direction and guidance. These align with negative past, which relates to a negative perspective on the past that may result from the experience of unpleasant events, the negative reconstruction of pleasant events, or a combination of both perspectives [16]. Both people with early maladaptive schemas and people with a negative past perspective have experienced a past full of unpleasant events. Therefore, people who have many early maladaptive schemas will also have a negative past perspective.

Early maladaptive schemas do not indirectly predict a negative past perspective through life expectancy. This issue can be analyzed so that although early maladaptive schemas directly predict a negative past perspective, since life expectancy could not predict a negative past perspective in this study, the indirect relationship between early maladaptive schemas and negative past perspective did not prove significant.

Early maladaptive schemas do not directly predict a positive past perspective. This lack of prediction can be accepted in such a way that impaired autonomy and performance is one of the domains of early maladaptive schemas. This domain includes schemas of dependence/incompetence, vulnerability to harm, undeveloped self/entanglement, and failure [18]. In this domain, the expectations that an individual has of themselves and their environment interfere with their tangible abilities for separation, survival, independent functioning, or successful task completion. Schemas in this domain typically emerge in families that diminish a child's self‐esteem, are intrusive, overly protective of the child, or have failed to encourage the child to engage in activities outside the family [18]. Impaired autonomy and performance domain in adulthood is correlated with the feeble bond between mother and child, both directly and through the mediation of expressive suppression [40]. All of these lead the individual to be heavily reliant on their family and naturally have fond memories and close relationships with family members, shaping these schemas. For example, in the undeveloped self/entanglement schema, we see an individual having an excessively emotional and close relationship with a significant person (often parents) [18]. All of these predict a positive past perspective because it originates from a bright and warm attitude toward the past, and psychologically, what individuals believe happened in the past influences their current thoughts, emotions, and behaviors more than what happened [41]. However, the remaining domains of early maladaptive schemas predict a negative past perspective, resulting in no meaningful relationships. Furthermore, early maladaptive schemas are not related to a positive past. Individuals usually have negative and bitter pasts upon which these schemas are formed, and they are silent about positive past perspective domains such as limitations or other quiet directions.

Early maladaptive schemas indirectly predict a positive past perspective. Early maladaptive schemas have a negative relationship with life expectancy and predict it negatively. Therefore, if a person has more schemas, the likelihood of having life expectancy decreases. This decrease in life expectancy distracts the individual from their positive past and brings negative thoughts to their mind, leaving no opportunity to cherish positive memories. However, suppose the number of schemas in an individual is low. In that case, life expectancy increases, and according to the eighth hypothesis of this study, which has been confirmed, a positive outlook is also predicted. Therefore, although there was no direct relationship between early maladaptive schemas and a positive past perspective, life expectancy acts as a mediator, and the indirect relationship becomes significant.

Early maladaptive schemas directly predict a hedonistic present perspective. The hedonistic present perspective encompasses a pleasure‐seeking approach and a willingness to take risks regarding time and life. The motto of these individuals in life is “I take risks to enjoy my life; I live for today and don't sacrifice it for tomorrow's rewards.” [16] Research indicates that individuals with a hedonistic present‐time perspective are more likely to engage in risky behaviors such as unprotected sexual relationships, substance abuse, and alcohol consumption [6]. These behaviors have a positive correlation with domains of limitations, self‐centeredness, and impaired functioning because within the subsets of these domains, individuals exhibit avoidance of responsibility, prevent conflict at all costs, and do not hold themselves accountable excessively [18], or they believe that they cannot handle everyday responsibilities such as taking care of others, solving daily problems, making sound judgments, coping with new duties, and making decisions without significant help from others, at an acceptable level [18]. Alternatively, the underdeveloped self‐schema manifests as emptiness and confusion, leading to aimlessness and purposelessness [18].

Early maladaptive schemas indirectly predict a hedonistic present perspective. This is because when early maladaptive schemas become more prevalent, life expectancy decreases in the individual, and this decrease in life expectancy leads to a reduction in the hedonistic present perspective. Therefore, early maladaptive schemas indirectly predict a negative hedonistic present perspective.

Early maladaptive schemas directly predict a deterministic present perspective. Deterministic individuals have a pessimistic and resigned view of life and the future, lacking belief in change and decision‐making for the future [6]. This deterministic inclination is understandable among individuals who have lived in poverty for a long time and have faced failure in their attempts to improve their life circumstances [16]. These individuals can be observed in early maladaptive schemas such as failure, vulnerability to harm, underdeveloped self, distrust, abandonment, and others.

Early maladaptive schemas indirectly predict a deterministic present perspective. When early maladaptive schemas become more prevalent, life expectancy decreases in the individual, and this decrease in life expectancy leads to an increase in the deterministic present perspective. Therefore, early maladaptive schemas indirectly predict a positive deterministic present perspective.

Early maladaptive schemas do not directly predict a future perspective. This lack of prediction can be understood in that schemas within the domains of impaired autonomy and performance, and over‐vigilance/inhibition have a positive relationship with future perspective. In contrast, the remaining schemas have a negative relationship with future perspective. Therefore, it cannot be assumed that there is a relationship between early maladaptive schemas and future perspectives. Future‐oriented individuals are ambitious and driven by their goals. Their performance is within the scope of their lives, and their decisions are more long‐term and less short‐term [41]. Future‐oriented individuals are competitive and dynamic, experiencing significant time pressure. They sacrifice family, friends, and much happiness for success [6]. For example, these traits are associated with the over‐vigilance/inhibition schema, where there is a fundamental belief that one must exert significant effort to meet high standards of behavior and performance to prevent criticism, which typically arises in families under pressure, excessively critical of themselves and others, and expect tasks to be completed with excellent quality in the shortest time possible. This schema often leads to severe deficiencies in pleasure, tranquility, health, sense of worth, progress, or satisfying relationships [18] and has a positive relationship. On the other hand, it has a negative relationship with the failure schema, which involves the belief that the individual has failed or will fail in the future and that failure is inevitable. Compared to their peers, individuals often feel incompetent in areas of progress such as education, career, sports, etc [18].

Early maladaptive schemas indirectly predict a future perspective. When early maladaptive schemas become more prevalent, life expectancy decreases in the individual, and this decrease in life expectancy leads to a reduction in future perspective. Therefore, early maladaptive schemas indirectly predict future perspective.

## 5 Conclusion

Based on the explanations provided for the research hypotheses and considering that the final model fit was confirmed, it can be concluded that the time perspective can be predicted based on early maladaptive schemas with the mediation of life expectancy. This means that early maladaptive schemas predict positively for the deterministic present, hedonistic present, and negative past dimensions. People who have more early maladaptive schemas are more likely to have deterministic present, hedonistic present, and negative past perspectives. early maladaptive schemas indirectly predict negatively for future and positive past dimensions through the mediation of life expectancy. Individuals with fewer early maladaptive schemas are more likely to have positive past and future perspectives.

This study is the first research that examines the relationship between time perspective and early maladaptive schemas. This study can be a start to further investigate the relationship between people's schemas and their way of looking at life and time. This study shows that children should achieve their basic needs so that maladaptive schemas are not formed in them, and if the needs are not met and maladaptive schemas are formed, then they will have negative effects on their view of life and time. For this purpose, training courses can be organized to tell parents about the importance of meeting the basic needs of children and to explain the important role of schemas in the formation of their children's personality and their time perspective for parents. Considering that this study shows that early maladaptive schemas lead to inappropriate time perspectives, therefore schema therapy may be effective for balancing people's time perspective, which requires another research. This study can be a start to expand schema therapy and use it to correct inappropriate time perspectives. This article points to a new benefit of schema therapy.

### 5.1 Study Limitations

Due to the nature of the study, causality cannot be established. Due to the lack of a list of students and the lack of university cooperation and the large number of samples, we had to use the convenience sampling method. This makes the results of this study not be generalizable to the whole society. For this study, we needed a large number of participants, that's why this study was conducted on students because students were available and the response rate was higher among students. Studying on students has made it impossible to generalize the results of this study to other populations, and it is good to conduct this issue in nonstudent populations, different populations and contexts in future research. Also, more accurate sample collection methods should be used.

## Author Contributions

**Mohammad Saeed Abedi Yarandi:** conceptualization, methodology, software, data curation, investigation, funding acquisition, validation, formal analysis, resources, visualization, writing – original draft, writing – review and editing. **Mohammad Ebrahim Madahi:** writing – review and editing, project administration, supervision. Preparation of questionnaires, data collection, data analysis and writing of the article were done by Abedi. The coordination of the ethics committee, university coordination, guidance and supervision were with Madahi.

## Ethics Statement

This study was approved by the Research Council of Shahed University and its ethical committee with the ethics code IR. SHAHED. REC.1402.030. In this study, students were entirely voluntary to participate.

## Conflicts of Interest

This study had no financial or moral sponsor. We thank Shahed University for allowing us to conduct this study there.

## Transparency Statement

The lead author Mohammad Saeed Abedi Yarandi affirms that this manuscript is an honest, accurate, and transparent account of the study being reported; that no important aspects of the study have been omitted; and that any discrepancies from the study as planned (and, if relevant, registered) have been explained.

## Data Availability

The data that support the findings of this study are available from the corresponding author upon reasonable request.
